# The Na/K-ATPase role as a signal transducer in lung inflammation

**DOI:** 10.3389/fimmu.2023.1287512

**Published:** 2024-01-17

**Authors:** Adriana Ribeiro Silva, Kauê Franscisco Correa de Souza e Souza, Thamires Bandeira De Souza, Mauricio Younes-Ibrahim, Patrícia Burth, Hugo Caire de Castro Faria Neto, Cassiano Felippe Gonçalves-de-Albuquerque

**Affiliations:** ^1^ Laboratório de Imunofarmacologia, Instituto Oswaldo Cruz, Fundação Oswaldo Cruz (FIOCRUZ), Rio de Janeiro, Brazil; ^2^ Laboratório de Imunofarmacologia, Departamento de Ciências Fisiológicas, Universidade Federal do Estado do Rio de Janeiro, Rio de Janeiro, Brazil; ^3^ Departamento de Biologia Celular e Molecular, Instituto de Biologia, Universidade Federal Fluminense, Niterói, Brazil; ^4^ Departamento de Medicina Interna, Faculdade de Ciências Médicas, Universidade do Estado do Rio de Janeiro, Rio de Janeiro, Brazil

**Keywords:** Na/K-ATPase, lung inflammation, ARDS, oleic acid, cardiac glycosides

## Abstract

Acute respiratory distress syndrome (ARDS) is marked by damage to the capillary endothelium and alveolar epithelium following edema formation and cell infiltration. Currently, there are no effective treatments for severe ARDS. Pathologies such as sepsis, pneumonia, fat embolism, and severe trauma may cause ARDS with respiratory failure. The primary mechanism of edema clearance is the epithelial cells’ Na/K-ATPase (NKA) activity. NKA is an enzyme that maintains the electrochemical gradient and cell homeostasis by transporting Na^+^ and K^+^ ions across the cell membrane. Direct injury on alveolar cells or changes in ion transport caused by infections decreases the NKA activity, loosening tight junctions in epithelial cells and causing edema formation. In addition, NKA acts as a receptor triggering signal transduction in response to the binding of cardiac glycosides. The ouabain (a cardiac glycoside) and oleic acid induce lung injury by targeting NKA. Besides enzymatic inhibition, the NKA triggers intracellular signal transduction, fostering proinflammatory cytokines production and contributing to lung injury. Herein, we reviewed and discussed the crucial role of NKA in edema clearance, lung injury, and intracellular signaling pathway activation leading to lung inflammation, thus putting the NKA as a protagonist in lung injury pathology.

## 1 Introduction

Acute respiratory distress syndrome (ARDS) is a lethal or disabling clinical syndrome triggered by sepsis, pneumonia, and severe trauma (1) with a high morbidity and mortality rate (2). The first report of patients with ARDS occurred in Denver, Colorado, in 1967 (3). According to the updated Berlin definition, ARDS is a syndrome with bilateral diffuse infiltrates on the chest followed by non-cardiogenic respiratory failure with mild, moderate, or severe oxygenation impairment (4). Also, ARDS pathology is characterized as damage to the capillary endothelium and alveolar epithelium with infiltrate accumulation in the alveolar space, forming interstitial and alveolar edema (5).

Fluid management is one of the most critical measures impacting ARDS, and dynamic monitoring of the lung fluid balance seems to influence clinical outcomes (6). Alveolar edema clearance depends on the vectorial transport of sodium and water across the alveolar epithelium. It happens, in part, through apically located sodium channels (ENaC) that drain the liquid into the lung interstitium via the basolaterally located Na/K-ATPase (NKA) and next to the capillary net through specific transcellular channels, as the aquaporins (7). Pathological changes in the alveolar-capillary barrier occur during pulmonary infection, altering NKA expression and ion channels in epithelial cells, causing edema and impairing alveolar fluid clearance (8).

Inhibitory NKA molecules are potential candidates to induce lung injury (9) because they block alveolar edema fluid clearance, which depends on the vectorial transport of sodium (7, 10). Molecules altering NKA activity may induce or increase lung edema and inflammation. Therefore, we link cell signaling induced by NKA to triggering lung inflammation.

## 2 Physiology of fluid transport in the lung/NKA as an ion transporter

The mammal’s respiratory system comprises the conductive and respiratory portions, including the respiratory bronchiole and alveoli (11). The nasal cavity is lined by pseudostratified columnar ciliated cells, the bronchioles lined by simple columnar, cuboidal epithelium, and the alveoli lined by a thin squamous epithelium (12) formed by squamous type I cells and cuboidal type II cells. Those cells are essential to homeostasis maintenance, antimicrobial control, host defense, pathogen recognition (13), and tissue repair (14).

**Figure 1:**
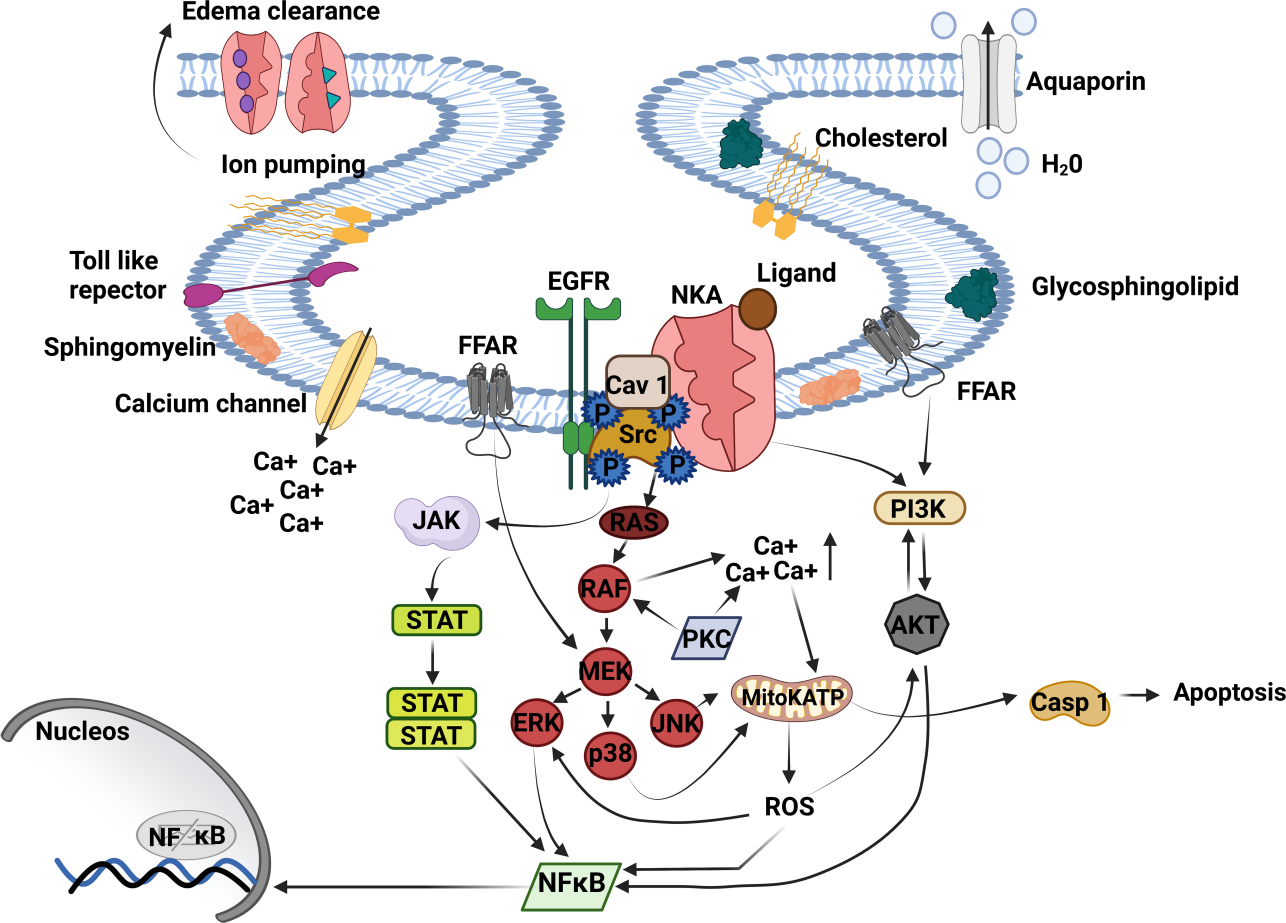
Na+/K+-ATPase signalosome. NKA acts as an ion pump and signal transducer, triggering lung inflammation. The binding of cardiac glycosides, GLP, oleic, and linoleic acid to NKA signalosome located in caveolae transduces signals to multiple pathways. The CG binding to NKA activates Src tyrosine kinase – EGFR complex is associated with inflammatory response and proinflammatory mediator production. Activated EGFR recruits’ protein adaptors that activate Ras - RAF (MAPK). RAF directly regulates MEK transactive JNK, p38, and ERK being the last activating NFκB. MAPK activation triggers the opening of mitochondrial ATP-sensitive potassium channels (mitoKATP) through increased intracellular Ca^++^, producing ROS production and casp 1 activation. ROS release stimulates ERK, AKT, and NFκB activation. Moreover, AKT can activate PI3K and NFκB or be phosphorylated by PI3K, essential to inflammation and edema formation. Another critical pathway that triggers lung injury is JAK/STAT3. JAK/STAT3 is regulated by Src upstream and ends with NFκB activation. NKA, Na^+^, K^+^ ATPase; Ca^++^, calcium; Src, non-receptor tyrosine kinases; EGFR, epithelial growth factor receptor; JAK, Janus kinase; STAT, signal transducer and activation of transcription; RAS, rat sarcoma virus; RAF, proto-oncogene serine; MAPK, mitogen-activated protein kinase; MEK, MAPK–ERK activating kinase; ERK, extracellular signal-regulated kinases; p38, mitogen-activated protein kinase; JNK, c-Jun n-terminal kinase; mitoKATP, mitochondrial ATP-sensitive potassium channel; ROS, reactive oxygen species; Casp-1, caspase-1; NFκB, factor nuclear kappa B; PI3K, phosphoinositide 3′ kinase; AKT, protein B kinase; FFAR, free fatty acid receptor. Created with BioRender.com.

The squamous type I cells are fragile and have large cytoplasmic extensions, ideal for gas exchange (15). Also, it plays an active role in water permeability and regulating alveolar fluid homeostasis through the NKA, ENaC, and water channels of the aquaporin 3 and 5 (AQP3 and AQP5) (16). Cuboidal type II cells play a crucial role in the lung immune response, participating in the tissue repair process after pulmonary injury, producing and secreting pulmonary surfactant, promoting transepithelial water movement, and expressing immunomodulatory proteins for host defense (15). In transepithelial water flux, these cells remove fluid from the alveolar space through sodium transport ions by NKA and ENaC channels (**Figure 1**) (17).

NKA maintains the electrochemical gradient and cell homeostasis (18) through ions across the cell membrane (19). Its structure is formed by three subunits, α subunit or catalytic (4 isoforms), β subunit or regulator (4 isoforms), and γ subunit (FXYD1-7 proteins) that is auxiliary to the holoenzyme complex with regulatory function (20). Like the NKA isoforms, each NKA subunit is expressed tissue- and cell-specific. For example, the α1 and β1 isoform NKA is the predominant isozyme expressed in alveolar epithelial cells type I and II, and the α2 subunit is present in alveolar epithelial type I cells (21). The β3 isoform is found in rat lungs, but its function remains unknown (22).

Alveolar cells NKA are located on the basolateral membrane and actively contribute to alveolar edema clearance (7). Additionally, ENaC and cystic fibrosis transmembrane conductance regulator (CFTR) are located apically (23, 24), contributing to edema clearance. Finally, the AQP5 transports water across the apical membrane in epithelial cells (16). However, the foremost mechanism of lung edema clearance is the transport of ion sodium and ion chloride across epithelial cells type I and II (7, 25), bringing water from the lungs into the capillaries (**Figure 1**).

## 3 NKA and tight junction integrity

NKA also has an essential role in regulation and the formation of tight junctions (26). Tight junctions are barriers between cells that control the passage of substances between alveolar and interstitial spaces through active transport (27, 28). Abnormal regulation of tight junction barrier allows the passage of pathogens and antigens through epithelial monolayers favoring disease development (28). Tight junctions also act as scaffolding platforms for cell signaling and docking stations for transport vesicles (29). Structurally, they comprise heteromeric occludin and claudin protein complexes that form a sealed interface between adjacent airway epithelial cells (30).

Together with the adapter proteins, the tight junctions can regulate tension, selective permeability, cell signaling, and gene expression (31, 32). However, insults such as lung injury (25), mechanical stress (33), fungal infection (34), viral and bacterial infection (35), and kinases activation (36) cause dysregulation tight endothelial junctions (37, 38) worsening lung disease.

The NKA β1 subunit overexpression upregulates tight epithelial junctions decreasing lung permeability independent of NKA pumping function (39). Also, NKA effects on tight junction formation depend on sodium concentration in the cell, preventing the formation of the stress-bundled fibers (40).

Sodium increase during NKA inhibition changes epithelial cells’ polarity (41). The disruption of tight junction complexes at the lung epithelial cells is deleterious and leads to lung edema formation in ARDS (42). In addition, establishing the tight junction drives epithelial polarization (43). Thus, the NKA influences the epithelial polarity, regulation, and tight junctions, strengthening the alveolar-epithelial barrier in a mechanism involving β1 subunit and myotonic dystrophy kinase-related cdc42-binding kinase α (39). Conversely, NKA inhibition leads to tight junctions’ integrity loss, increasing the alveolar permeability and worsening acute lung injury/ARDS.

## 4 Molecules that affect NKA and induce or amplify lung injury

Cardiac glycosides are known worldwide as classical inhibitors of the NKA (44). They were identified as a plant secondary metabolite, and later on as an endogenous mammalian substance produced by adrenal gland, hypothalamus, and hypophysis (45). The cardiac glycosides bind to the NKA α subunit, increasing the intracellular concentration of ions calcium and sodium (44), resulting in reactive oxygen species (ROS) production due to intracellular signaling activation (46). Also, they inhibit ion-pumping (7), decreasing edema fluid clearance (8). The cardiac glycoside concentration directly impacts NKA’s effect, triggering cell signaling at low concentrations and blocking the ionic pump at high concentrations (47). Our group recently reviewed the role of NKA in cell adhesion, motility, and migration in cancer cells (48). Cardiac glycosides induce apoptosis and autophagy in transformed cells, involving molecular pathways inducing deleterious effects on lung cancer cells (48). In addition, different cardiac glycoside concentrations can also have pro- or anti-inflammatory effects on lung (45).

In 1985, Tamura and co-workers showed that free unsaturated fatty acid such as linoleic acid (LA) and oleic acid (OA) in plasma inhibits NKA promoting increased extracellular fluid accumulation in hogs (49). Also, OA inhibits the NKA *in vitro* (50, 51). Furthermore, our group and others showed that lung NKA inhibition induces lung injury and an inflammatory microenvironment in the lungs (52–56). Ouabain, a cardiac glycoside, induced lung injury in mice (52). In addition, OA *in vivo* causes lung injury with immune cell accumulation, edema formation, inflammatory mediators production that amplify inflammatory response, such as tumor necrosis factor alfa (TNF-α), interleukin 1 beta (IL-1β), leukotriene B_4_-(LTB_4_), and intracellular pathways activation ((mitogen-activated protein kinase (MAPK), phosphoinositide 3’ kinase (PI3K), extracellular signal-regulated kinase 1/2 (ERK1/2), factor nuclear kappa B (NF-κB)) in lung tissues (56, 57). Thus, these data reinforce the importance of NKA activity for lung edema clearance and make it a potential molecular target for lung injury treatment (**Figure 1**).

Changes in the alveolar–capillary barrier happen during pulmonary infection and cause altered expression of epithelial NKA, decreasing alveolar fluid clearance. TGF-β, TNF-α, interferons, or IL-1β are produced after infection with *Streptococcus pneumoniae, Klebsiella pneumoniae, Mycoplasma pneumoniae*, influenza A virus, pathogenic coronaviruses, or respiratory syncytial virus and amplify alveoli-capillary disturbances and damage (8).

## 5 NKA as a receptor and signal transducer

Cardiac glycosides activate the signalosome, a NKA protein complex restricted to the caveolae, triggering intracellular signaling pathways (19). The cell signaling from NKA occurs through the interaction of signalosome proteins such as NKA, caveolin-1, non-receptor tyrosine kinases (Src), rat sarcoma virus protein (RAS) binding of small guanosine triphosphate (GTP) (19), and epidermal growth factor receptor (EGFR) (58). EGFR is a transmembrane protein with cytoplasmic kinase activity that stimulates cell proliferation, differentiation, growth, and migration (59). Caveolin-1, an integral protein cell membrane, interacts with several tyrosine kinases, like EGFR, and connects Src kinase in the MAPK pathway activation. Src belongs to the Src family kinase (SFKs) (60). The caveolin-1 forms a spontaneous complex with NKA and interacts with Src kinase forming the active NKA-caveolin-1-Src **Figure 1** (58).

In addition, Src kinases and NKA act together to transduce outside-in signaling by ouabain (19). Then, Src binds to the α-subunit of NKA and triggers the Src/EGFR-MAPK pathway signaling (61). Src activation affects different cellular processes such as adhesion, migration, cell differentiation (60), and ROS formation (61). EGFR activates Ras-proto-oncogene serine (Raf)-MAPK, PI3K (59), which increases ROS production (62), NFκB activation, and proinflammatory cytokine production (63). Activation of the PI3K pathway by ouabain can also result in the endocytosis of the NKA-Src complex (19), blocking NKA signaling in the myocyte. Also, ouabain triggers EGFR-Src-Ras-Raf-MAPK–ERK activating kinase (MEK)-extracellular signal-regulated kinases (ERK1/2) pathway and Src-independent activation of PI3K1A and Akt, leading to fibroblast proliferation (**Figure 1**) (64).

The cardiac glycosides activate Src and trigger the ERK 1/2 signaling through the Ras-Raf-MEK pathway-independent intracellular calcium fluctuations (65). ERK activation induces the production of proinflammatory mediators and can be activated by MEK-MAPK (66), EGFR (67), and ROS (68). Also, EGFR transactivation by Src activates ERK 1/2 through the activation of Ras-Raf-MEK cascade (69). In addition, the MAPK activation induces an increase in calcium concentration, favoring the opening of mitochondrial adenosine triphosphate (ATP)-sensitive K^+^ channels, which increases nicotinamide adenine dinucleotide phosphate (NADPH) oxidase activity and generates mitochondrial ROS (70).

The specific NKA cell signaling is related to disease etiology (71). Despite several studies denoting the link between NKA and intracellular signaling pathways activation (72), some details in the signaling cascade remain to be unveiled.

## 6 NKA in signal transduction during lung injury

Summarizing what has been introduced before, the NKA activity on edema fluid clearance is crucial to lung injury (7, 9). The impairment of the alveoli-capillary barrier and damage to the alveolar epithelium occur during acute lung injury and ARDS, resulting in the accumulation of protein-rich edema fluid (73), impairing gas exchange (7). Epithelium cells type I and II, through NKA activity, remove salt and water from the alveoli (74). Furthermore, cell signaling from NKA of the epithelium cells type I and II activate proinflammatory pathways in the lung, which is harmful to the lung. Lung injury happens by inhibiting or triggering intracellular signaling. Interestingly the ion-pumping function increases due to NKA cell signaling activation (75). In addition, cardiac glycosides, OA, and LA in the lung inhibit NKA and induce injury through NKA signaling cascade activation (55, 76) (77) (**Figure 1**).

Besides cardiac glycosides, FXYD proteins modulate NKA activity. FXYD5 overexpression in mice damages the alveoli-epithelial barrier and causes lung inflammation. LPS stimulation of alveolar epithelial cells and mice lungs increased FXYD5 plasma membrane expression, NF-κB activation, and cytokine production. FXYD5-deficient cells have not responded to LPS. FXYD5 overexpression increased the monocyte migration to the lung, and in turn, FXYD5 silent mice showed less CCL2, monocytes, and protein extravasation after LPS stimulation. As it occurred *in vitro*, the FXYD5 effect depended on NF-κB, and they were not just involved in the LPS-inflammatory effect and cytokines, reinforcing the role of a NKA modulator in inflammation (78).

NKA activates Src and ERK. Activating Src and ERK leads to lung injury and inflammation (79). Src activates ERK signaling (80) and induces proinflammatory cytokine production (81). Also, neutrophil migration, edema, lipid body formation, and cytokine IL-6 production in lung injury depend on ERK activation in an OA-induced lung injury animal model (57). Furthermore, JNK, p38, p65 (77), and p50 phosphorylation (82) is linked to a decrease of lung alveolar permeability and neutrophil accumulation in the bronchoalveolar lavage fluid (BALF). Inhibiting those pathways reduces lung vascular leak and suppresses the NF-kB pathway (82).

ARDS patients that express high angiotensin-converting enzyme (ACE) in BALF have Ras phosphorylated (83, 84). Furthermore, it suggested that Ras protein regulates pulmonary vascular tone, which can contribute to the pathogenesis of ARDS (84). Raf and MEK phosphorylation and the release of IL-1β, IL-4, IL-6, and TNFα are increased in LPS-induced acute lung injury (85). In addition, RAF activates JNK and p38 and ERK, activating NF-κB, contributing to increased lung permeability and inflammation in ARDS (86).

The signaling of cardiac glycosides occurred through the NKA/α-1-Src kinase complex in immune cells to produce ROS and inflammatory cytokines. NKA α-1 knock-down or the specific inhibition of the NKA α-1-Src kinase complex by pNaKtide, or even the Src inhibitor PP2 impaired the inflammatory effect of cardiac glycoside telecinobufagin (TCB) in macrophages. TCB, ouabain, digoxin, and marinobufagenin activated NF-κB via NKA α-1-Src kinase complex (87).

PI3K, a NKA downstream signaling protein, is essential in lung inflammation and edema (88). Its inhibition may alleviate lung injury with lower leukocyte accumulation and edema formation in the lungs (56). PI3K, through AKT activation, regulates broad cellular processes, including apoptosis, proliferation, and differentiation (89).

Different pathways activated by NKA signaling are similar to LPS-triggered ones and crosstalk with pathways triggered by LPS during lung cell and immune cell stimulation. Ouabain activated NF-κB, leading to proinflammatory cytokine synthesis (MCP-1/CCL-2, TNF-α, IL-1β, and IL-6) by murine peritoneal macrophages and human monocyte-derived macrophages. Macrophages partially deficient in NKA lacked ouabain-induced NF-κB activation, and consequently the synthesis of proinflammatory mediators (90). Ouabain in fact may have either pro or anti-inflammatory role (76, 91, 92). Ouabain challenged the monocytes to produced IL-1β, TNF-α, IL-10 and VEGF, showing its immunomodulatory role (92). Ouabain also inhibits proinflammatory monocyte activation, downregulating membrane CD14 and CD16 in early time points (91). Others showed NKA binding of cardiac glycosides leads to IL-1β production and release. The mechanisms of the cardiac glycoside digoxin and others include activation of the NLRP3 inflammasome in macrophages at concentrations used in clinical treatment. The result is the induction of the programmed cell death pathway through caspase-1, called pyroptosis. Which causes inflammation and tissue damage showing the direct correlation of NKA signaling to the induction of IL-1β via inflammasome (93). In turn, inhibiting inflammasome activity reduced ischemic damage (94). IL-1β expression in the lungs is associated with neutrophil recruitment and induction of acute lung injury (95), acting as a biomarker of barrier integrity loss in the lungs via interaction with EGFR and claudins (96). Also, IL-1β stimulates the IL-6 and TNF-α release (97). The claudins are proteins that form intercellular junctions supporting the permeability and ion selectivity of paracellular pores of the large airways (98). Among them, cldn-18 is highly expressed in the alveolar epithelium, while cldn-4 is vital in regulating paracellular permeability during alveolar fluid clearance supporting the resolution edema (25). In addition, IL-18 controls the release of IL-1β (97), being both (IL-1β and IL-18) activated by caspase-1 (97), which is critical to the development of sepsis/ARDS (99).

The leptospiral endotoxin glycolipoprotein (GLP) binds to NKA and causes organ dysfunctions during leptospirosis. Metabolic alterations increase free fatty acid levels in the blood and lipotoxicity. During the infection, inflammasomes are formed, and NKA once again leads to pro-inflammatory and metabolic alterations and involves inflammasome activation. NKA is related to the severity and considered a therapeutic target (100).

Activation of NKA triggers JAK/STAT3 pathway signaling, an inductor of lung injury (101, 102). STAT activation induces IL-6 release (103), and mutation in STAT3 impairs IL-6 activity and recurrent infections in the lungs (104). Also, IL-6 and TNF-α release are regulated by JNK (105) and p38 (77, 106). The p38 causes deleterious effects and cell death (107). When p38 is activated, the lung neutrophils induce tissue damage through the secretion of ROS and IL-8 (108), promoting endothelial and epithelial barrier dysfunction (109), and worsening ARDS. ROS upregulates the expression of proinflammatory cytokines and adhesion molecules, amplifying the tissue damage and pulmonary edema (109). Once released, IL-8 decreased cell viabilityand inhibited the expression of surfactant proteins A and B (33). The absence of surfactant protein in the lung increases the surface tension, resulting in the alveolar and peripheral airway collapse (110). TNF-α also downregulates the surfactant protein (111), potentializes the release of chemokines and cytokines, increases lung vascular permeability, and modulates the recruitment and activation of neutrophils, contributing to lung injury. NKA signaling activation may induce multiple signaling cascades that may cause lung injury and cause the production of proinflammatory mediators by structural cells and recruited leukocytes that amplify the inflammatory response in the lung (**Figure 1**). Further research may unveil additional molecular mechanisms and other receptors involved in the lung injury processes.

Fatty acids, such as the NKA ligands LA and OA, also bind to free fatty acid receptors (FFARs). Four FFARs have physiological importance in biological processes (FFAR1, FFAR2, FFAR3, and FFAR4, formerly known as GPR40, 43, 41, and 120) (112, 113). The long-chain free fatty acid (FFA) preferentially binds to FFAR1 and FFAR4, while short-chain fatty acid binds to FFAR2 and FFAR3 (114). Also, FFARs are similarly expressed in metabolic tissues and immune cells, regulating energetic metabolism and inflammatory responses (115). The FFARs exhibit overlapping functions through signaling pathways involving the activation of Ca^+2^, cAMP, or ERK1/2 responses, with G protein-dependent or independent pathways acting more as modulators rather than initiators of biological processes (116). For instance, OA generates an aggressive phenotype in prostate cancer cells via the PI3K/Akt signaling pathway depending on FFA1/GPR40 (117).

Non-Esterified Fatty Acids (NEFA) are FFA with elevated levels in obese individuals, and long-chain FFAs act as endogenous ligands of the FFAR1. OA, LA, and GW9508 (FFAR1/FFAR4 dual agonist) induced human airway smooth muscle cell proliferation, dependent on p70S6K phosphorylation through MEK/ERK and PI3K/Akt signaling pathways (118). Saturated fatty acids (SFA) are thought to reduce vascular reactivity by decreasing insulin signaling via vasodilator pathways (PI3K/Akt/endothelial nitric oxide synthase (eNOS)) and enhancing pro-inflammatory pathways. In comparison, OA promotes signaling via the PI3K/Akt/eNOS pathway (119). Fatty acids induced IKKβ activity, with palmitic acid showing greater activation than OA and LA, reducing nitric oxide production (120). FFAR1 and FFAR4 are expressed in the lungs (113). FFAR4 is highly expressed in murine lungs and appears to be restricted to the airway epithelium, mainly on mucous-secreting goblet cells and ciliate columnar epithelial cells (121, 122). Saturated and unsaturated long-chain fatty acids may bind FFAR1 and FFAR4 and activate intracellular signaling, such as PI3K, ERK, and PKC (116, 123). Thus, FFAR1 and FFAR 4 activation may activate intracellular pathways with crosstalk with NKA signalosome.

Interestingly, FFAR4 activation promotes bronchial epithelial repair after epithelial injury, and this event possibly occurs by fatty acid-specific induction through FFAR4 (124). High levels of FFAR4 expression in the lungs will be linked with airway function and dysfunction (123), so the specific type of FFA (saturated, unsaturated, or polyunsaturated) that binds and activates FFAR4 may have a positive or a negative impact on lung injury.

## 7 Conventional treatments and NKA as a possible therapeutic target

Several preventive therapies were tested unsuccessfully in patients with lung injury at the initial stages aiming to prevent acute lung injury and the progression of ARDS (125). Previous studies showed the effectiveness of the treatments based on exogenous surfactant, inhaled nitric oxide, intravenous prostaglandin E1, glucocorticoids, ketoconazole, lisofylline, n-acetylcysteine, and activated protein C. However, effectiveness could not be confirmed when these potential treatments were advanced to phase III clinical studies (126). Therefore, new therapeutic approaches have been suggested for acute lung injury/ARDS treatment, and NKA has been indicated as a possible therapeutic target (127). Some studies had already signaled the edema clearance improvement through stimulation of NKA activity by aldosterone (128), growth factors (129), catecholamines (130, 131), β-adrenergic agonist (132), β-adrenergic receptor overexpression (133), dopamine (134), vasopressin (135) and rosuvastatin (127). This effect is expected because NKA of alveolar epithelial cell types I and II support edema clearance (136).

On the other hand, gene therapy studies with the overexpression of the NKA β_1_ subunit upregulated the expression of tight junction proteins, leading to increased alveolar epithelial barrier function (39). Machado-Aranda and coworkers (2005) tested gene therapy using electroporation of the NKA β_1_ subunit to increase alveolar fluid reabsorption (137). Similarly, a study with transfection with adenoviruses carrying genes encoding the α_1_ and the β_1_ subunit of NKA showed a decrease of ARDS in C57/BL6 mice (138). Additionally, the gene transfer of the NKA α_2_ subunit by adenovirus also increased NKA activity in rat alveolar epithelial cells and adenocarcinoma human alveolar basal epithelial cells (A549 cells) and improved the basal lung fluid clearance rate (139).

The importance of other ionic channels for edema clearance, such as ENaC (140) and AQPs (141) has also been demonstrated in ARDS. Although important, ENaC and AQPs are not essential for the alveolar fluid removal (142, 143), conversely, the NKA which is identified as the primary edema clearance agent (143). Therefore, new therapeutic approaches, including gene therapy targeting NKA, can be an effective alternative to improve edema clearance, favoring lesion recovery in acute lung injury and ARDS

## 8 Concluding remarks

Acute lung injury/ARDS is a respiratory failure syndrome marked by the disruption of alveolar endothelial and epithelial barriers and accumulation of edema fluid within the alveolus and interstitium (73). Edema clearance is crucial for resolving these lung injuries and can be accomplished by NKA activity in type I and II epithelial cells. Nevertheless, NKA may trigger lung injury through signaling pathways independent of ion pumping inhibition. Interestingly, the Src-EGFR-Ras-Raf-MAPK signaling cascade is the main NKA pathway and the responsible for triggering lung injury, fostering neutrophil influx into the lungs (79), edema and lipid body formation, NF-κB transactivation (63), IL-1β, IL-4, IL-6, TNFα and ROS production (57, 144). Therefore, NKA is a protagonist in edema clearance but may also induce lung injury by triggering cell signaling independent of its pumping activity.

Additionally, the overexpression of the NKA β1 subunit avoids lung injury. This occurs because of the improvement in edema clearance by NKA pumping activity, decreasing alveolar fluid accumulation. Besides, the integrity of the alveolar epithelial barrier between alveolar and interstitial spaces depends on the NKA activity. Further studies are necessary to fully elucidate the specific pathways triggered by NKA on lung injury and develop alternative therapeutic strategies targeting NKA for acute lung injury/ARDS treatment.

## Author contributions

AS: Conceptualization, Funding acquisition, Writing – original draft, Writing – review & editing. KSS: Writing – original draft. TS: Writing – original draft. MY-I: Funding acquisition, Writing – original draft. PB: Conceptualization, Funding acquisition, Writing – original draft, Writing – review & editing. HCFN: Conceptualization, Funding acquisition, Writing – original draft, Writing – review & editing. CG-d-A: Conceptualization, Funding acquisition, Supervision, Writing – original draft, Writing – review & editing.
